# Whole Genome Expression Analysis in a Mouse Model of Tauopathy Identifies MECP2 as a Possible Regulator of Tau Pathology

**DOI:** 10.3389/fnmol.2017.00069

**Published:** 2017-03-17

**Authors:** Nicole M. Maphis, Shanya Jiang, Jessica Binder, Carrie Wright, Banu Gopalan, Bruce T. Lamb, Kiran Bhaskar

**Affiliations:** ^1^Department of Molecular Genetics and Microbiology, University of New Mexico, AlbuquerqueNM, USA; ^2^Lieber Institute for Brain Development, BaltimoreMD, USA; ^3^Department of Biostatistics, Cleveland Clinic Foundation Cleveland OH, USA; ^4^Stark Neurosciences Research Institute, Indiana University, IndianapolisIN, USA

**Keywords:** Alzheimer’s disease, tauopathies, tau protein, tau transgenic mice, methyl-CpG-binding protein-2, MECP2, microarray

## Abstract

Increasing evidence suggests that hyperphosphorylation and aggregation of microtubule-associated protein tau (MAPT or tau) correlates with the development of cognitive impairment in Alzheimer’s disease (AD) and related tauopathies. While numerous attempts have been made to model AD-relevant tau pathology in various animal models, there has been very limited success for these models to fully recapitulate the progression of disease as seen in human tauopathies. Here, we performed whole genome gene expression in a genomic mouse model of tauopathy that expressed human *MAPT* gene under the control of endogenous human *MAPT* promoter and also were complete knockout for endogenous mouse tau [referred to as ‘hTau^*MaptKO*(*Duke*)^′ mice]. First, whole genome expression analysis revealed 64 genes, which were differentially expressed (32 up-regulated and 32 down-regulated) in the hippocampus of 6-month-old hTau^*MaptKO*(*Duke*)^ mice compared to age-matched non-transgenic controls. Genes relevant to neuronal function or neurological disease include up-regulated genes: PKC-alpha (*Prkca*), MECP2 (*Mecp2*), STRN4 (*Strn4*), SLC40a1 (*Slc40a1*), POLD2 (*Pold2*), PCSK2 (*Pcsk2*), and down-regulated genes: KRT12 (*Krt12*), LASS1 (*Cers1*), PLAT (*Plat*), and NRXN1 (*Nrxn1*). Second, network analysis suggested anatomical structure development, cellular metabolic process, cell death, signal transduction, and stress response were significantly altered biological processes in the hTau^*MaptKO*(*Duke*)^ mice as compared to age-matched non-transgenic controls. Further characterization of a sub-group of significantly altered genes revealed elevated phosphorylation of MECP2 (methyl-CpG-binding protein-2), which binds to methylated CpGs and associates with chromatin, in hTau^*MaptKO*(*Duke*)^ mice compared to age-matched controls. Third, phoshpho-MECP2 was elevated in autopsy brain samples from human AD compared to healthy controls. Finally, siRNA-mediated knockdown of MECP2 in human tau expressing N2a cells resulted in a significant decrease in total and phosphorylated tau. Together, these results suggest that MECP2 is a potential novel regulator of tau pathology relevant to AD and tauopathies.

## Introduction

Tauopathies are a class of neurodegenerative diseases characterized by the accumulation of hyperphosphorylated, oligomeric and aggregated tau protein as NFTs (Lee et al., 2001). A classic example of tauopathy is AD, which is the most common neurological disease in the elderly leading to dementia. In addition to AD, PSP, CBD, PiD, and FTDP-17 are other examples of pure neurodegenerative tauopathies (Lee et al., 2001), where dysfunctional tau is the primary driver of the disease. In a majority of these neurodegenerative tauopathies, the non-mutant form of tau has been shown to be the primary causative factor. However, in FTDP-17, single point mutations in either the splice-site or coding region of the *MAPT* gene has shown to impair the structure and function of tau, inducing behavioral, cognitive, and psychological impairments (Hasegawa, 2006). A growing number of studies implicate reasonably significant positive correlations between the build-up of NFTs and cognitive impairment in various tau-targeted imaging studies in humans (Nelson et al., 2012; Murray et al., 2015; Brier et al., 2016; Ossenkoppele et al., 2016; Scholl et al., 2016). Although it is still unclear how exactly hyperphosphorylated and/or aggregated tau contributes to neurodegenerative processes, numerous studies have suggested that tau-mediated pathological events occur either via loss-of-function or gain-of-toxic function with regards to microtubule interactions. Altering signaling pathways, like Src/Fyn (Lee et al., 2004; Bhaskar et al., 2005; Vossel et al., 2010; Morris et al., 2011), somato-dendritic localization affecting synaptic function (Ittner et al., 2010; Ittner and Gotz, 2011), impairing neurogenesis (Komuro et al., 2015), neuron-to-neuron propagation and seeding of NFTs (Liu et al., 2012), as well as contributing to neuroinflammatory responses (Zilka et al., 2012) are just a few of the suggested tau-mediated pathological events.

Over the past several years, tau pathology has been replicated in several animal models of tauopathy that carry familial mutations (Gotz et al., 2007). However, the tau pathology observed is often in ectopic locations (Lewis et al., 2000) and/or without overt neuronal cell death in these models (Gotz et al., 2007). Davies and colleagues developed a genomic mouse model of human tau pathology (HTau) expressing the entire human non-mutant *MAPT* gene (Duff et al., 2000) in a partial mouse *Mapt* deficient background (Andorfer et al., 2003). Several previous studies have established that HTau mice exhibit age-related hyperphosphorylation and aggregation of MAPT (Andorfer et al., 2003), accumulation of NFTs and displayed significant neuronal cell death (Andorfer et al., 2005). While HTau mice displayed age-related tau pathology and neurodegeneration, it was not clear if there was any influence by the endogenous mouse tau, as the *Mapt* knockout mice utilized to generate these HTau mice still retained 31 amino acids in the N-terminal end of tau (Tucker et al., 2001). This is an important factor to consider as we have previously demonstrated that the N-terminal region of tau, which includes Tyr18, is a substrate for the Src family non-receptor tyrosine kinase (SFKs, for example: Fyn) (Lee et al., 2004) and can regulate SFK signaling (Sharma et al., 2007) probably via interaction between pTyr18 of tau with SH2 domain of SFKs. In a separate study, Dawson et al. (2001) generated a separate *Mapt^-/-^* mice line and crossed them to transgenic mice expressing human *MAPT* to generate hTTg^+/^*^-^*/*Mapt^-^*^/^*^-^* mice. Primary neurons derived from hTTg^+/^*^-^*/*Mapt^-^*^/^*^-^* mice displayed restoration of total axonal length and total length of minor processes (compared to *Mapt^-^*^/^*^-^* mice) *in vitro.* However, these studies were done primarily in cell culture and further characterizations of these mice are yet unavailable.

In the current study, we crossed HTau/*Mapt^+/+^* mice [line 8C (Duff et al., 2000)] with the complete mouse *Mapt* knockout mice (Dawson et al., 2001) to generate hTau^*MaptKO*(*Duke*)^ and identify novel gene(s) differentially regulated exclusively by the expression of human *MAPT* via whole genome gene expression analysis using Illumina^®^ WG6 microarray analysis. We further assessed the protein levels of differentially expressed gene(s) and their activation (phosphorylation) states in hTau^*MaptKO*(*Duke*)^ mice and also in human AD brains. Finally, we performed siRNA-mediated knockdown studies to determine the relationship between a newly identified gene, MECP2, in regulating tau levels in *in vitro* model of tauopathy.

## Materials and Methods

### Mice

The HTau (Andorfer et al., 2003) mice (expressing human *MAPT* with partial deficiency for endogenous mouse *Mapt*, was generated via targeted insertion of EGFP into the *Mapt* locus hence referred to as *Mapt^egfp^*^/^*^egfp^*) were bred and maintained in our colony. Mice with a complete knockout of endogenous mouse *Mapt* (Dawson et al., 2001) (*Mapt^-/-^)* were obtained from Jackson Laboratory (B6.129X1-*Mapt^tm1Hnd^*/J – Stock # 007251). Both HTau and *Mapt^-^*^/^*^-^* mice were maintained in the C57BL/6 background. We outbred HTau to the C57BL/6J (non-transgenic) mice to obtain the 8c line (HTau mice with *Mapt*^+/+^ background). The HTau/*Mapt*^+^*^/^*^+^ were then crossed to *Mapt^-/-^* mice (Dawson et al., 2001) over at least 10 generations to develop hTau-*Mapt*^-^*^/^*^-^ mice, which will be referred to as hTau^*MaptKO*(*Duke*)^ mice [‘*MaptKO(Duke)*’ refers to ‘*Mapt* knockout mice generated at the Duke University by Dawson’s group [published in Dawson et al. (2001) study]. All comparisons were made to either 6- or 12-month-old non-transgenic mice (referred to as WT). The WT mice were littermates generated during hTau^*MaptKO*(*Duke*)^ and *Mapt*^-^*^/^*^-^ breeding, but were without human *MAPT* transgene and were *Mapt^+/+^.* We utilized mixed gender for all groups of mice in the present study (**Table 1**). All experimental protocols involving animals were performed in accordance with the US National Institute of Health guidelines on animal care and were approved by the Institutional Animal Care and Use Committee of the University of New Mexico and Cleveland Clinic Foundation.

**Table 1:** Sample description.

Sample #	Sample name	Sample description	Sex	Sample group	Sentrix ID	Sentrix position
1	WT1	6-month-old WT	M	NM052412A	6189772009	A
2	WT2	6-month-old WT	F	NM052412B	6189772009	B
3	WT3	6-month-old WT	F	NM052412C	6189772009	C
4	hTau1	6-month-old hTau^*MaptKO*(*Duke*)^	F	NM052412D	6189772009	D
5	hTau2	6-month-old hTau^*MaptKO*(*Duke*)^	F	NM052412E	6189772009	E
6	hTau3	6-month-old hTau^*MaptKO*(*Duke*)^	M	NM052412F	6189772009	F


### Antibodies and Reagents

*Microtubule-associated protein tau antibodies:* AT8 (pS202/pT205; mouse monoclonal antibody; ThermoFisher Scientific; #MN1020), AT180 (pT231; mouse monoclonal antibody; ThermoFisher Scientific; #MN1040), Tau-5 (or Tau5; mouse monoclonal antibody; ThermoFisher Scientific; #AHB0042), PHF-1 (pS396/pS404; mouse monoclonal antibody provided by Peter Davies, Albert Einstein College of Medicine), Tau12 (human tau specific antibody; mouse monoclonal antibody; Abcam; #ab74137). *Other antibodies:* GAPDH (rabbit polyclonal antibody; EMD Millipore; #ABS16), T-MECP2 (D4F3; rabbit monoclonal antibody; Cell Signaling #3456); phospho-MECP2 (pSer80; rabbit polyclonal antibody; PhosphoSolutions #p1205-80).

### Tissue Preparation for Biochemical Analysis

The mice were anesthetized and transcardially perfused with 0.125 M phosphate buffer (PB). Following perfusion, the brains were removed, the left hemisphere was immersion fixed in 4% paraformaldehyde in PB (4% PFA/PB), the right hemisphere was micro-dissected into the cortex and hippocampus, wet weights were recorded, and the tissues were snap frozen in liquid nitrogen for subsequent biochemical analysis. The rest of the right hemispheres were weighed and snap frozen in liquid nitrogen for subsequent mRNA extraction. Hippocampi from 6- and 12-month-old WT and hTau^*MaptKO*(*Duke*)^ mice were used for biochemical (quantitative real-time PCR/qRT-PCR or Western blot) analysis. Sagittal brain sections were used for the immunohistochemical analysis (see below).

### RNA Extraction, Whole Genome Microarray, and Gene Expression Analysis

**FIGURE 1:**
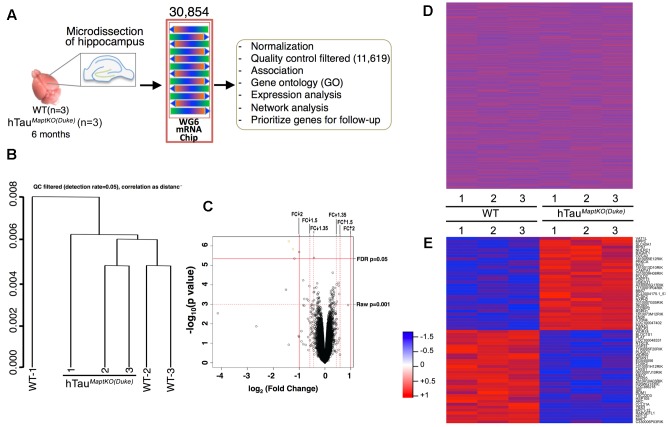
**Sample processing and cluster analysis approach.** **(A)** Hippocampi from both hemispheres of 6-month-old non-transgenic (WT) and hTau^*MaptKO*(*Duke*)^ mice (*n* = 3 per genotype) were isolated, total RNA purified and profiled for 30,854 transcripts via MouseWG-6 v2.0 Expression BeadChip Kit (Illumina^®^), and the data was processed for downstream analysis. **(B)** Hierarchical clustering (unsupervised) with correlation distance (QC filtered detection rate = 0.05) show WT-2 and WT-3 clustered together while WT-1 stayed separate. All three hTau^*MaptKO*(*Duke*)^-1-3 clustered together or close to each other. **(C)** Volcano plot shows the relationship between –log10 (*p*-value) and log2 (fold change, FC) of all the genes being compared between WT and hTau^*MaptKO*(*Duke*)^ groups. Note the two criteria used in identifying the altered genes; (1) FDR adjusted *p*-value < 0.05; (2) Raw *p*-value < 0.001; up and down arrow depict up- or down-regulated genes, respectively. **(D)** Expression intensities of all 30,854 normalized genes in the hippocampi of 6-month-old WT (*n* = 3) and 6-month-old hTau^*MaptKO*(*Duke*)^ (*n* = 3) mice, represented by red and blue, for high and low intensities, respectively. **(E)** Expression intensities of 64 genes that were significantly altered in hTau^*MaptKO*(*Duke*)^ mice compared to WT mice. Intensity scale is shown on the left of **(E)**.

RNA from the hippocampus of 6-month-old non-transgenic (or WT) and hTau^*MaptKO*(*Duke*)^ mice (**Table 1**) was extracted using the TRIzol^®^ reagent as described by the manufacturer (Thermo Fisher Scientific), quantitated and 3 μg (30 μl at 100 ng/μl) of purified RNA converted to cRNA (High Capacity cDNA synthesis kit, Thermo Fisher Scientific) and hybridized on Illumina^®^ Mouse WG6 microarray chip (Pool_ID: 11278593_A; Sentrix_ID: 6189772009; see the layout in **Table 2**). The Illumina Mouse WG6 chip was a single chip that contains six whole genome microarrays. Each of these six microarrays on the WG6 chip contained 30,854 oligonucleotides (**Figure 1A**; corresponding to over 23,000 well-characterized genes and over 10,000 predicted genes). Microarray chips were scanned using Illumina’s state-of-the-art iScan^®^. The raw data from iScan^®^ were pre-processed in GenomeStudio and exported for further data processing and analysis in R-LIMMA package (Law et al., 2016).

**Table 2:** Conditions and samples distribution.

**Conditions**	**Chip1**		
	
WT	1	2	3
hTau^*MaptKO*(*Duke*)^	4	5	6


*The samples were not evenly distributed across chips.*

For the gene expression analysis: Total RNA (50 ng/μL) was converted to cDNA using the High Capacity cDNA Reverse Transcription kit and amplified using specific TaqMan probes (*gene expression marker: Mecp2* Mm01193535_m1; *MAPT* Hs00902194_m1) GAPDH was used as a house-keeping gene for normalization. The qPCR assays were run on the StepOnePlus^®^ Real-Time PCR System (all reagents were purchased from Thermo Fisher Scientific).

### SDS-PAGE and Western Immunoblotting

Proteins were homogenized in 10% weight/volume Tissue Protein Extraction Reagent (T-PER^®^, Thermo Fisher Scientific) and soluble hippocampal lysates were resolved via SDS-PAGE and immunoblotted as previously described (Bhaskar et al., 2010b). The dilutions of primary antibodies utilized were as follows: GAPDH at 1:20,000; Tau5 at 1:10,000; phospho- and total-MECP2 at 1:1000.

### Immunohistochemical Analysis

Free-floating sections (30 μm thick) from 12-month-old WT and hTau^*MaptKO*(*Duke*)^ mice as well as formalin fixed autopsy brain sections from cognitively normal (Braak stage 0) and AD (Braak Stage V) were processed for immunohistochemical (IHC) and immunofluorescence (IF) analysis as previously described (Bhaskar et al., 2010b). Briefly, sections were first incubated in 10 mM sodium citrate buffer (pH 6.0) for 10 min at 95°C for antigen retrieval, washed in PBS with 0.1% Tween (PBST), quenched with 0.3% H_2_O_2_ in PBST for 15 min (only for IHC). Sections were blocked for 1 h at room temperature with the 5% normal sera (goat/donkey – from animal species in which the secondary antibodies were raised). The sections were incubated with phospho-MECP2 (1:250). After washing in PBST, the sections were incubated with biotinylated-secondary antibody (1:250; Jackson ImmunoResearch Laboratories Inc., for IHC) or Alexa 555 conjugated goat anti-rabbit secondary antibody (1:250 in blocking solution; for IF). Sections were then incubated with ABC (Vector Laboratories) reagent for 1 h at room temperature (for IHC). The immunoreactive signals were revealed by developing sections in SigmaFast^®^ 3,3’-diaminobenzidine (DAB) tablets (Sigma–Aldrich) (for IHC) or the sections were mounted on slides using Hardset (Vector Laboratories) with DAPI (for IF). Bright field images were acquired using Leica DMR upright fluorescence, Leica TCS-SP8 confocal microscope and FLIM system or Olympus bright field microscope.

### Cell Lines and Transfections

Mouse neuroblastoma cells (Neuro2a or N2a cells; ATCC^®^, Manassas, VA, USA) were cultured in EMEM with L-glutamine media with 10% FBS for up to 10 passages before being expanded and plated at a density of 500,000 cells per well for all the experiments. N2a cells were transiently transfected with human tau plasmid (0.4 μg DNA per transfection), pRC/CMV n123c (0N3R tau) using Effectine^®^ reagent per manufacturer’s instructions (Qiagen, Cat# 301425). Mock (empty plasmid) or tau transfected N2a cells were nucleofected with siRNA to MECP2. After 24 h, the cells were lysed to detect MECP2, PHF1, Tau5, Tau12, β-actin, and GAPDH via SDS-PAGE and Western blot analysis (see above). Ratios of MECP2/GAPDH were plotted to assess MECP2 knockdown and ratios of PHF1/Tau5 and Tau5/GAPDH were plotted and quantified.

### Statistical Analysis

Unless otherwise indicated, comparisons between the two groups were done via unpaired *t-*test; comparisons between multiple treatment groups were done via one-way or two-way ANOVA with indicated multiple comparisons *post hoc* tests. All statistical analyses were performed using GraphPad Prism^®^.

## Results

The goal of the current study was to perform a whole genome gene expression analysis in the humanized mouse model of tau pathology that expressed only human tau in the complete mouse tau knockout background. The expectations were two fold; (1) to identify novel gene(s) that were significantly altered in response to only human tau expression; and (2) to correlate those significantly altered gene(s) with tau hyperphosphorylation in hTau^*MaptKO*(*Duke*)^ mice and in human tauopathy.

To obtain a genomic-based human mouse model of tauopathy, we first outbred Htau mice [developed by Dr. Peter Davies (Andorfer et al., 2003) to C57Bl/6J non-transgenic mice and obtained human tau expressing mouse line called ‘8c’ (Duff et al., 2000)]. The 8c line expresses all six isoforms of human *MAPT* transgene driven by the endogenous human *MAPT* promoter. The line 8c was then bred to the complete mouse *Mapt*^-^*^/^*^-^ mice, which were previously reported by Dawson et al. (2001). The strategy for *Mapt* gene disruption utilized insertion of a NEO cassette in place of exon 1 of the *Mapt* gene which resulted in the complete replacement of exon 1 by NEO cassette and lead to the total loss of endogenous mouse tau (Dawson et al., 2001). We called these hTau (8c line) mice with complete deficiency of endogenous mouse *Mapt* as hTau^*MaptKO*(*Duke*)^ mice [‘*MaptKO(Duke)*’ refers to *Mapt*^-^*^/^*^-^ mice generated by Dawson et al. (2001), from Duke University (published in Dawson et al. (2001) study)]. These hTau^*MaptKO*(*Duke*)^ mice are humanized genomic mice, which express all six isoforms of human tau driven by human *MAPT* promoter and are homozygous-deficient for endogenous mouse *Mapt*.

### A Small Number of Genes Are Differentially Expressed in the Hippocampus of 6-Month-Old hTau^*MaptKO*(*Duke*)^ Mice Compared to Age-Matched Non-transgenic Mice

To identify differentially expressed mRNA levels in the hippocampus of 6-month-old WT and hTau^*MaptKO*(*Duke*)^ mice (*n* = 3 per genotype; **Table 1**), we used an Illumina^®^ MouseWG-6 V2 array chip to probe changes in 30,854 different mRNAs (**Figure 1A**). A 6-month time point was selected based on, the results from present study and several previous studies where Htau mice have been shown to display significant increase in tau phosphorylation in the hippocampus (Andorfer et al., 2003, 2005; Maphis et al., 2015). Overexpression of human *MAPT* in hTau^*MaptKO*(*Duke*)^ mice did not alter the overall number of mRNA messages detected in the hippocampus between WT mice and hTau^*MaptKO*(*Duke*)^ mice that were relative to all the probes present on the array (data not shown).

This primary whole genome microarray screen, which was based on a small group size, resulted in generation of three data sets: (1) the raw expression dataset exported directly from GenomeStudio, referred to as raw data; (2) the normalized dataset, which was obtained based on the raw dataset after force-positive background correction, log_2_ transformation and quantile normalization; (3) the QC filtered dataset, which was obtained based on the normalized dataset by selecting the present probes using the detection threshold of *p* < 0.05. The raw and normalized datasets had 30,854 genes and the QC filtered dataset had 11,619 genes (**Figure 1A**). Next, QC analysis was also performed on the normalized expression data and the results are plotted as normalized box plot, normalized control, normalized M (log ratios) and A (mean average) scale plot, normalized pairs, and normalized sample relation (Supplementary Figures S1–S4). Overall, the QC analysis of the normalized data suggested that the generated dataset was of good quality for the downstream analysis. Next, hierarchical clustering (unsupervised) was performed on the three data sets. Correlation distance was used in all cases. Results suggested some association between sample conditions and clustered grouping (Supplementary Figure S5). For example, samples from WT-2 and WT-3 (non-transgenic mice #2 and #3) segregated together. Similarly, samples from hTau^*MaptKO*(*Duke*)^-2 and hTau^*MaptKO*(*Duke*)^-3 segregated together. However, samples from WT-1 and hTau^*MaptKO*(*Duke*)^-1 were separate from other samples in their respective groups (**Figure 1B** and Supplementary Figure S5). Overall, there were no outliers in the sample sets after the QC and clustering analysis.

Next, comparisons were performed via linear modeling to check differences between the two genotypes [WT and hTau^*MaptKO*(*Duke*)^]. Empirical Bayes and other shrinkage methods were used to borrow information across genes. FDR adjusted *p*-values were calculated. Genes were defined as significantly altered, if their FDR-adjusted *p*-value < 0.05. We also calculated raw *p-*value and fold change. For the initial screening purposes, the differentially altered genes were defined as those genes whose adjusted *p*-value < 0.05 or raw *p*-value < 0.001 whichever was smaller, and whose fold change was greater than the threshold or less than 1/threshold in this analysis (Illumina^®^ recommends 1.35 for the threshold). A volcano plot analysis was performed which showed the relationship between –log_10_ (*p-*value) and log_2_ (fold change) of all the genes between WT and hTau^*MaptKO*(*Duke*)^ groups. Genes scattered beyond the cut-off window in the volcano plot indicated the statistical significance of gene expression changes and provided a direct visual representation of the genes that were differentially expressed. Based on the fact that only four genes (*Gh, Krt12, Mapt*, and *Rapgefl1*; **Table 3**) were altered between WT and hTau^*MaptKO*(*Duke*)^ mice when we used a strict FDR cut-off (of 2.0), we relaxed our criteria to include genes that met both the raw *p*-value and fold change (FDR cut-off of 1.5) (**Figure 1C**). Prior to relaxing our criteria the heat-map analysis of all 30,854 genes showed no obvious pattern of significantly altered genes between WT and hTau^*MaptKO*(*Duke*)^ groups (**Figure 1D**). However, following relaxation of our criteria (with a raw *p*-value of <0.001), 64 genes were either up- or down-regulated in hTau^*MaptKO*(*Duke*)^ mice compared to WT group (**Figure 1E** and **Table 3**) and only these were utilized for downstream functional pathway annotation and network analysis via IPA^TM^, Metacore^TM^, and GO (**Table 3**). Some of the differentially regulated genes, proteins they encode, their function and their relevance to the brain function are shown in Supplementary Table S1.

**Table 3:** Description of gene selection for pathway analysis.

Total genes studied	30,854
QC filtered genes	11,618
Significantly altered [all are down-regulated in hTau^*MaptKO*(*Duke*)^ mice] genes in hTau^*MaptKO*(*Duke*)^ vs. WT mice (Based on raw *p*-value 10^-6^ and FDR cut-off < 0.05)	4 (*Gh, Krt12, Mapt*, and *Rapgefl1*)
Genes after relaxing the statistical criteria (Raw *p*-value of <0.001)	64
Number of genes used for GO, IPA^TM^, Metacore^TM^ studies	62 (2 were obsolete)


### A Majority of the Altered Genes Are Regulated by the Transcription Factor SP1 and Are Associated with Anatomical Structure, Development, Metabolic Process, and Cell Death

**FIGURE 2:**
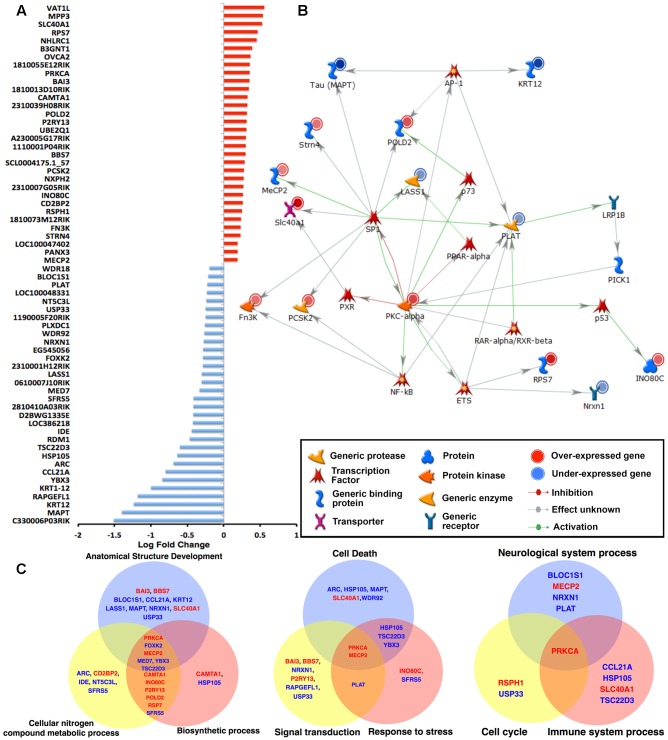
**Microarray analysis of the hippocampi of 6-month-old WT and hTau^*MaptKO*(*Duke*)^ mice.** **(A)** Total RNA from hippocampi of 6-month-old WT and hTau^*MaptKO*(*Duke*)^ mice was hybridized to MouseWG-6 v2.0 Expression BeadChip (Illumina^®^). Expression of 64 genes that were significantly altered in hTau^*MaptKO*(*Duke*)^ mice compared to WT is shown. Values displayed as fold change in expression level: up-regulated (red) and down-regulated (blue) genes. Data represents mean fold change from three mice per genotype. **(B)** Gene interaction network analysis (using Metacore analytical suite) for the 64 significantly altered genes in hTau^*MaptKO*(*Duke*)^ mice compared to WT mice. The genes with red (or blue) next to their graphic key is either up- (or down-) regulated. As expected, endogenous mouse tau (MAPT) is one of the most down-regulated genes. Other genes relevant to neuronal function or neurological disease include *Prkca, Mecp2, Strn4, Slc40a1, Pold2, Pcsk2* (up-regulated) and *Krt12, Lass1, Plat* and *Nrxn1* (down-regulated). Many of the altered genes are regulated by the transcription factor SP1. **(C)** Venn diagrams showing GO’s biological processes when all 64 genes are categorized for top biological processes segregate into three distinct Venn diagrams: Group 1: anatomical structure development – 17 altered genes out of total 47, i.e., 17/47; cellular nitrogen compound metabolic process – 15/47; biosynthetic process 13/47. Group 2: cell death – 9/47; signal transduction 8/47; response to stress 8/47. Group 3: neurological system 5/47, cell cycle 2/47 and immune system processes 4/47. Note the genes written in red (or blue) are significantly up- (or down-) regulated.

To obtain an inventory of all the up- and down-regulated genes between WT and hTau^*MaptKO*(*Duke*)^ mice, we plotted all 64 significantly altered (raw *p* < 0.001) genes displaying a cut-off of +1.5-fold change (**Figure 2A**). Interestingly, exactly half of the 64 genes (i.e., 32) were down-regulated and the remaining half was up-regulated (**Figure 2A**). The most down-regulated gene was *C330006P03RIK*, which is an unclassified gene of unknown function. The most up-regulated gene, yet with only ∼0.5 log fold change, was *Vat1L* protein, which is a protein-coding gene with functions related to oxidoreductase activity and transferase activity (source: GeneCards and GO annotations) (**Figure 2A**). *Mecp2* and *Wdr18* were among the lowest (<0.25-fold) up- or down-regulated genes, respectively (**Figure 2A**). While *Mecp2* was the gene with the lowest level of upregulation in hTau^*MaptKO*(*Duke*)^ mice, it is highly relevant to our analysis because of its involvement in the regulation of DNA methylation. Furthermore, a mutation in the *MECP2* gene in humans is the primary cause of most cases of Rett Syndrome (Joyner et al., 2009; Petazzi et al., 2014), a rare genetic postnatal neurological disorder predominantly affecting females. Next, we performed network analysis using Metacore^TM^ to identify which transcription factor(s) would regulate the expression of the most number of genes from our screening. As shown in **Figure 2B**, SP1 transcription factor appeared to regulate 9 of the significantly altered genes (*Mapt, Strn4, Mecp2, Slc40a1, Fn3K, Pcsk2, Prkca, Lass1*, and *Pold2*) in our panel compared to other transcription factors: NFκB, PXR, ETS, RAR-α/RXR-β, p53, p73 or AP-1, which only appeared to regulate anywhere between 1 and 4 genes (**Figure 2B**).

Next we performed process and phenotype annotations for the genes that are significantly altered in the hTau^*MaptKO*(*Duke*)^ mice using Metacore^TM^, IPA^TM^, and GO. There were overlaps in certain functional terms between these analyses. For example, the enrichment analysis from these publicly available software packages showed that the following process/functions were pronounced in hTau mice: “Regulation of protein modification and metabolic processes, acetylation, protein-protein interaction, protein ubiquitination, regulation of apoptosis, cell surface receptor linked signal transduction,” etc., while “alternative splicing, metal binding, presence of WD40 motif proteins, secretory pathway” were relatively suppressed in hTau^*MaptKO*(*Duke*)^ compared to WT. The protein modification, ubiquitination, protein transport, cytoskeletal remodeling, inflammation, etc., were some of the key process/function terms that surfaced from the IPA/Metacore analysis. To identify the most relevant set of genes that overlapped with more than one functional GO terminology, and the set of genes that would be most relevant to neurological diseases, we plotted the top six biological process categories from the GO analysis and mapped genes that were up- (red) or down-regulated (blue) within each of these biological processes. The top six GO biological process and the gene representations were; (a) anatomical structure development (17/47 genes; 36.17%); (b) cellular nitrogen compound metabolic process (15/47 genes, 31.91%); (c) biosynthetic process (13/47 genes, 27.66%); (d) cell death (9/47 gene, 19.15%); (e) signal transduction (8/47 genes, 17.02%); and (f) response to stress (8/47 genes; 17.02%) (**Figure 2C**). Differentially altered genes associated with these six biological processes were split into two groups and plotted as two Venn diagrams (**Figure 2C**). The intersection included *Mecp2*↑ and *Prkca*↑ (arrows indicate these are up-regulated) as key genes common for both sets of Venn diagrams. The overlapping region also included *Foxk2*↓, *Med7*↓, *Ybx3*↓, and *Tsc22d3*↓ (arrows indicate these are down-regulated). However, these were absent in the second set of Venn diagram (**Figure 2C**). We also plotted a third Venn diagram with ‘neurological system process’ (5/47 genes; 10.64%), ‘immune system process’ (5/47 genes; 10.64%), and ‘cell cycle’ (3/47 genes; 6.38%) as three additional biological/disease-relevant processes. While *Prkca* was the only gene present in the overlapping region, *Mecp2* was clearly unique to ‘neurological system process’ (**Figure 2C**). Taken together, these results suggest that *Prkca* and *Mecp2* are the most common genes relevant to several biological processes, which may have been altered in the hippocampus of hTau^*MaptKO*(*Duke*)^ mice compared to WT controls.

### *Mecp2* Is Significantly Altered in hTau^*MaptKO*(*Duke*)^ Mice and Human AD Brain

**FIGURE 3:**
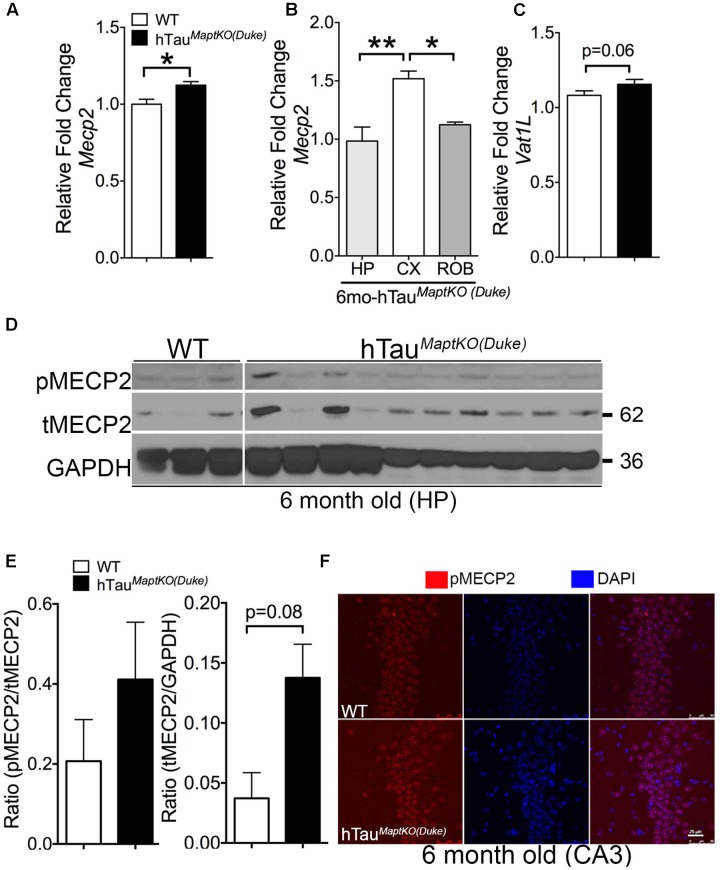
**MECP2 expression and phosphorylation is up-regulated in 6-month-old hTau^*MaptKO*(*Duke*)^ mice.** **(A)** qRT-PCR analysis showing statistically significant (^∗^*p* < 0.05; unpaired *t-*test; *n* = 4 WT, and *n* = 4 for hTau^*MaptKO*(*Duke*)^ mice; mean + SEM) up-regulation of MECP2 in the hemi-brains of 6-month-old hTau^*MaptKO*(*Duke*)^ vs. WT mice. **(B)** Note the regional differences in the expression of MECP2 in the hippocampus (HIP), cortex (CX), and rest of the brain (ROB) that are devoid of CX and HP. **(C)** Another gene (*Vat1l*) that was increased in our whole genome microarray analysis also showed modest up-regulation in its mRNA levels in the brains of hTau^*MaptKO*(*Duke*)^ mice compared to age-matched WT mice. **(D,E)** Western blot analysis showing a trend toward increased levels for phospho(p)-Ser80 MECP2/total (t) MECP2 and tMECP2/GAPDH [*p* = 0.08; unpaired *t-*test; *n* = 3, all females for WT and *n* = 10, three females and six males for hTau^*MaptKO*(*Duke*)^] in 6-month-old hTau^*MaptKO*(*Duke*)^ versus WT mice; mean + SEM). **(F)** Double IF and confocal microscopy analysis revealing a modest increase in pMECP2 in the CA3 region of HP in 6-month-old hTau^*MaptKO*(*Duke*)^ mice compared to age-matched WT controls. Scale bar 25 μm.

Whereas *Prkca* is a generic protein kinase that affects multiple cellular pathways, the *Mecp2* is one of the altered genes present in the interface of all three biological processes from the GO analysis and is directly implicated in neurological diseases (Rett Syndrome). Therefore, we decided to determine the total protein levels and phosphorylation status of MECP2 in the hippocampus of non-transgenic and hTau^*MaptKO*(*Duke*)^ mice. To confirm our microarray data, we first assessed *Mecp2* mRNA levels via real-time quantitative PCR analysis and observed an expected increase in the MECP2 mRNA levels in the hemi-brains of 6-month-old hTau^*MaptKO*(*Duke*)^ mice compared to age-mated WT controls (**Figure 3A**). Next, we determined the regional differences in the MECP2 mRNA levels with hTau^*MaptKO*(*Duke*)^ mice at 6 months of age. Interestingly, we observed cortex displaying significantly highest levels of MECP2 mRNA compared to hippocampus and rest of the brain (ROB), which are enriched in striatum, diencephalic structures, brain stem but lacked cortex/hippocampus (**Figure 3B**). We also assessed the mRNA levels of *Vat1l*, which was up-regulated in the microarray analysis. There was an increased trend for *Vat1l*, but the statistical significance was at *p* = 0.06 (**Figure 3C**). Next, we prepared hippocampal lysates from 6-month-old WT and hTau^*MaptKO*(*Duke*)^ mice. The levels of both phosphorylated at Ser80 (p)MECP2/total (t)MECP2 and tMECP2/GAPDH appeared elevated in the hippocampus of 6-month-old hTau^*MaptKO*(*Duke*)^ mice compared to 6-month-old WT controls (**Figures 3D,E**). However, only the total (t)MECP2 levels appeared increased more than three folds compared to WT controls (**Figures 3D,E**), yet statistical analysis suggested *p* = 0.08 despite having *n* = 10 mice in hTau^*MaptKO*(*Duke*)^ group (**Figures 3D,E**). This could be due to high variability in the levels of both pMECP2 and tMECP2 at 6-months of age in both WT and hTau^*MaptKO*(*Duke*)^ groups. Finally, we performed double IF analysis for pMECP2 and DAPI to determine if the pMECP2 levels reveal elevated labeling within specific cellular population in the hippocampus. Both 6-month-old WT and hTau^*MaptKO*(*Duke*)^ mice showed basal level of, mostly nuclear, labeling for pMECP2, which appeared slightly increased as a heterochromatic speckles within the nucleus of the CA3 region of hTau^*MaptKO*(*Duke*)^ mice compared to WT mice (**Figure 3F**). Together these results suggests that because the MECP2 mRNA levels are only marginally up-regulated in the whole genome gene expression and mRNA analysis, the MECP2 protein levels did not reach desired statistical significance in the hippocampus of hTau^*MaptKO*(*Duke*)^ mice.

**FIGURE 4:**
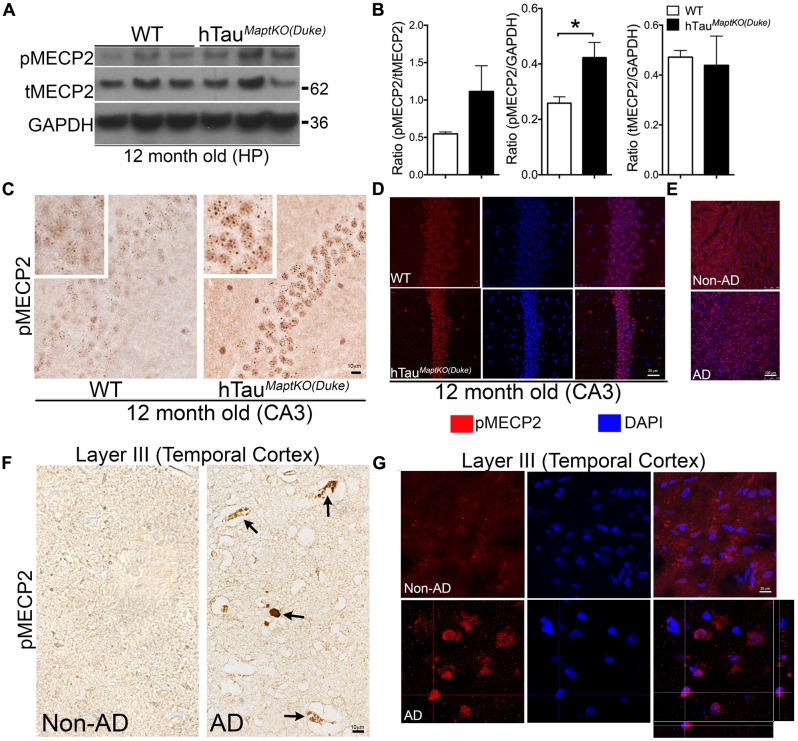
**MECP2 phosphorylation is up-regulated in 12-month-old hTau^*MaptKO*(*Duke*)^ mice and in human AD brain.** **(A,B)** Western blot analysis showing pMECP2/GAPDH ratio significantly higher [^∗^*p* < 0.05; unpaired *t-*test; *n* = 3 for hTau^*MaptKO*(*Duke*)^ versus WT mice; all three males for WT; two males and one female for hTau^*MaptKO*(*Duke*)^; mean + SEM] in the hippocampus of 12-month-old hTau^*MaptKO*(*Duke*)^ mice compared to WT controls. No alteration in the tMECP2/GAPDH ratio in the hippocampus of 12-month-old hTau^*MaptKO*(*Duke*)^ mice compared to controls. **(C)** Significantly elevated pMECP2 immunoreactive specks within the nucleus of CA3 hippocampal neurons of 12-month-old hTau^*MaptKO*(*Duke*)^ mice compared to age-matched WT controls. **(D)** Double IF and confocal microscopy analysis shows a modest increase in the pMECP2 in the CA3 neuronal layer of 12-month-old hTau^*MaptKO*(*Duke*)^ mice compared to age-matched WT controls. **(E–G)** Confocal projections (in **E,G**) and bright field images show elevated pMECP2 immunoreactivity and co-localization with nuclear stain DAPI (in **E,G**) or peri-vascular labeling (in **F**) specifically in the Layer III of temporal cortex of human AD brain autopsy sections compared to the to non-AD healthy control subject. Orthogonal view in **(G)** shows pMECP2 labeling to be nuclear or peri-nuclear in the human AD cortex. Scale bar 10 μm (in **C,F**); 25 μm (in **D,G**); 100 μm (in **E**).

To determine if the differences in MECP2 between WT and hTau^*MaptKO*(*Duke*)^ mice stands out to be statistically significant with age, we performed Western blot analysis in 12-month-old WT and hTau^*MaptKO*(*Duke*)^ mice. There was no significant alteration in the levels of T-MECP2 protein at 12-months of age (**Figures 4A,B**). However, the level of pMECP2 was significantly up-regulated in the hippocampus of hTau^*MaptKO*(*Duke*)^ mice compared to WT mice (**Figures 4A,B**). Elevated levels of pMECP2 were also observed within the nuclei of CA3 pyramidal neurons in hippocampus of hTau^*MaptKO*(*Duke*)^ mice compared to WT group, which again appeared more like speckle staining mostly at heterochromatin regions of the nucleus (**Figure 4C**). We confirmed this by performing double IF followed by confocal microscopy analysis. Twelve-month-old hTau^*MaptKO*(*Duke*)^ mice displayed markedly elevated pMECP2 labeling that overlapped with nuclear staining DAPI in the CA3 regions of hippocampus (**Figure 4D**). Finally, to determine if the pMECP2 is relevant to human AD, we probed autopsied brain sections from a human AD patient and compared that from a non-demented control subject. Confocal microscopy analysis revealed numerous cells positive for pMECP2 in the layer III (temporal cortex) of human AD brain compared to non-AD controls (**Figure 4E**). Interestingly, we observed significantly more pMECP2 immunoreactive structures, which appeared to be peri-vascular and picnotic (**Figure 4F** arrows) and sometimes nuclear/peri-nuclear in layer III of the temporal cortex (**Figures 4F,G**). Confocal microscopy did show presence of somewhat cytosolic or diffused nuclear staining for pMECP2 in non-AD controls (**Figure 4G**), which was readily visible in the IHC via bright field microscopy (**Figure 4F**). These results reveal higher levels of pMECP2 in both the CA3 hippocampal neurons of hTau^*MaptKO*(*Duke*)^ mice and in the temporal lobe of human AD patients.

### *Mecp2* Regulates the Levels of Total and Phosphorylated Tau Protein

**FIGURE 5:**
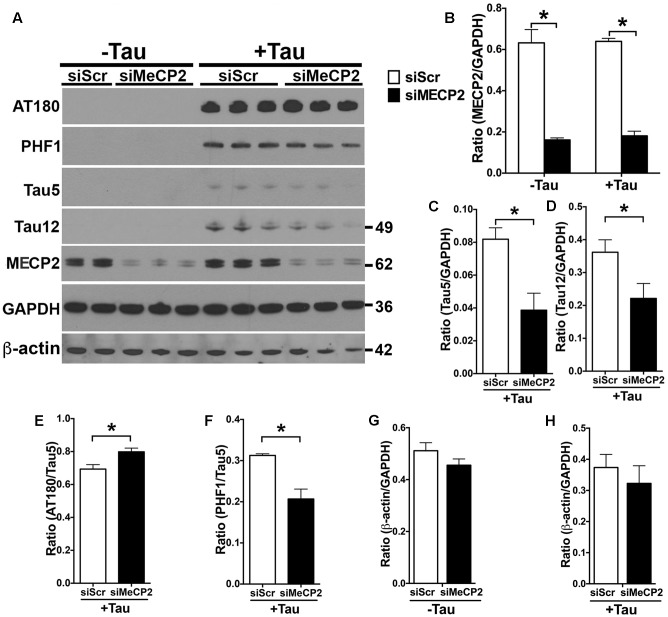
**MECP2 regulates tau pathology *in vitro*.** N2a cells transiently transfected with human tau 0N3R isoform (‘+Tau’) or a control plasmid (‘-Tau’) were nucleofected with siRNA [scramble siRNA (siScr) or MECP2 siRNA]. After 24 h of siRNA nucleofection, the cells were harvested and probed for AT180, PHF-1, Tau5, Tau12, and T-MECP2. **(A,B)** Note that siMECP2 significantly reduced levels of MECP2 in both ‘-Tau’ and ‘+Tau’ N2a cells (^∗^*p* < 0.01; unpaired *t-*test; *n* = 3 replicates; mean + SEM). **(C,D)** siMECP2 treatment also significantly (^∗^*p* < 0.01; unpaired *t-*test; *n* = 3 replicates; mean + SEM) reduced the levels of both total tau (Tau5/GAPDH) and human tau (Tau12/GAPDH) ratios in the ‘+Tau’ N2a cells compared to scramble siRNA treated conditions. **(E–H)** siMECP2 knockdown resulted in statistically significant (^∗^*p* < 0.01; unpaired *t-*test; *n* = 3 replicates; mean + SEM) increase and decrease in AT180/Tau5 and PHF1/Tau5 ratios, respectively. Note that the ratio for β-actin/GAPDH was not altered either in ‘-Tau’/‘+Tau’ conditions or with/without siMECP2 conditions.

Since the MECP2 levels are elevated in hTau^*MaptKO*(*Duke*)^ mice and in human AD, we wanted to determine if MECP2 could directly regulate the total and phosphorylated levels of tau protein. Therefore, we transiently transfected N2a cells with either human tau (non-mutant 0N3R isoform) or control plasmid. After 24 h, the N2a cells were nucleofected with either MECP2 siRNA (siMECP2) or scrambled siRNA (siScr). As expected, human tau transfected N2a cells expressed human tau (positive for Tau12 antibody) and displayed hyperphosphorylation on the T231 (AT180) and Ser396/Ser404 (PHF1) sites (**Figures 5E,F**). Further, siRNA against MECP2 showed >80% reduction in the T-MECP2 levels compared to siScr control group (**Figures 5A,B**). Interestingly, both hyperphosphorylated (on PHF1 site) and total tau (Tau5/GAPDH and Tau12/GAPDH) levels were significantly reduced following siRNA-mediated knockdown of MECP2 in tau transfected N2a cells (**Figures 5C,D**). On the other hand, most intriguingly, there was an increase in AT180/Tau5 ratio with siRNA-mediated knockdown of MECP2 was observed (**Figures 4A**, **5E**). To determine if MECP2 knockdown specific to reducing tau levels or could act as a suppressor of generic transcription; we assessed the immunoreactivity for β-actin. While there was small reduction in β-actin/GAPDH ratio in with siMECP2 in both ‘+/- Tau’ conditions (**Figures 5A,G,H**), however, the effects were not statistically significant as the one observed for Tau5/GAPDH and Tau12/GAPDH (**Figures 5A,C,D**). Together, these results suggest that MECP2 may directly or indirectly regulate expression of tau and its phosphorylation state, which may be relevant to AD and related tauopathies.

## Discussion

We report here on the generation and use of a new line of human tau transgenic mice (hTau^*MaptKO*(*Duke*)^) via crossing two previously reported mouse lines [line 8c – (Duff et al., 2000) and complete mouse tau knockout line (Dawson et al., 2001)]. We performed a whole genome gene expression analysis in the hippocampus of hTau^*MaptKO*(*Duke*)^ mice and compared it with age-matched control (WT) mice. Our results identified a small number of genes differentially expressed in hTau^*MaptKO*(*Duke*)^ mice compared to non-transgenic control groups. Among them *Mecp2* was highly relevant to neurological conditions such as Rett syndrome [reviewed in (Feldman et al., 2016)] and Autism (Loat et al., 2008). While T-MECP2 levels were not specifically altered in the hippocampus of aged hTau^*MaptKO*(*Duke*)^ mice, the active form (phosphorylated at Ser80) of MECP2 was significantly elevated in the hTau^*MaptKO*(*Duke*)^ compared to age-matched control mice. Finally, siRNA-mediated deficiency of MECP2 resulted in altered phosphorylation of tau (on AT180 and PHF1 sites) and significantly altered total tau levels in an N2a cell culture model of tauopathy, indicating an regulatory relationship between MECP2 and tau phosphorylation.

Our goal to generate hTau^*MaptKO*(*Duke*)^ mice with a complete deficiency of endogenous mouse tau was prompted by the lack of genomic models available where the endogenous *MAPT* promoter drives the expression of human *MAPT* in the *Mapt* null genetic background. The choice of such mouse model allows identification of differentially expressed genes due to the exchange of human tau against mouse tau. This could be of interest since tauopathies including AD are specific to humans and therefore might be related to specific feature of human tau compared to mouse tau. While the HTau mice developed by Davies and colleagues did display all the pathological features of tauopathies, endogenous *Mapt* deficiency was incomplete. Since these HTau mice retained the first 31 amino acids at the N-terminus of endogenous mouse *Mapt* and previous studies from our group (Lee et al., 2004; Bhaskar et al., 2005, 2010a; Sharma et al., 2007) and others (LaPointe et al., 2009; Pooler and Hanger, 2010) have demonstrated important functions for the N-terminal part of tau, we generated hTau^*MaptKO*(*Duke*)^ mice on a complete mouse *Mapt* null background for whole genome gene expression analysis. Furthermore, because the human *MAPT* is driven by endogenous human *MAPT* promoter, any differentially regulated genes in hTau^*MaptKO*(*Duke*)^ mice would more closely relate to the human condition than those from cDNA based mouse models of tauopathy – many of these carry mutations in *MAPT* gene and may only be relevant to FTDP-17. While numerous genome-wide association (GWAS) studies have been performed in AD and have identified several risk alleles, single nucleotide polymorphism (SNPs), and/or haplotypes, there are very limited GWAS studies conducted in human populations investigating pure tauopathy. Recently, the first PSP GWAS identified three non-*MAPT* susceptibility loci at *STX6, EIF2AK3*, and *MOBP* (Hoglinger et al., 2011). In another recent GWAS study in CBD cases, additional association of the *SOS1* and *lnc-KIF13B-1* along with overlapping associations of *MOBP* to both PSP and CBD were discovered (Kouri et al., 2015). Three additional candidate genes *ABCA7, DYSF*, and *PAXIP1* showed association in another recent multi-ancestral GWAS study of AD, FTD, and PSP (Chen et al., 2015). The most recent GWAS study in a population of PSP suggested that brain levels of *LRRC37A4* and *ARL17B* were associated with rs8070723; *MOBP* with rs1768208 and both *ARL17A* and *ARL17B* with rs242557 (Allen et al., 2016). Interestingly, authors discovered strong association between risk alleles and CpG methylation in several-associated genes/loci including *MAPT, MOBP*, *ARL17A*, and *ARL17B* (Allen et al., 2016). When we performed heat-map analysis for the normalized dataset for some of the PSP/CBD associated genes (*PAXIP1, SOS1, DYSF, EIF2AK3, STX6, ABCA7, MOBP*, and *MAPT*) in our whole genome screen, we noticed a very clear segregation of hTau^*MaptKO*(*Duke*)^ and WT mice (Supplementary Figure S6). Most importantly, *MOBP* segregated with *MAPT* and *ABCA7, while STX6* was closer to *MAPT/MOBP* (Supplementary Figure S6). The common link between our study and previously published human GWAS studies is the role of epigenetics in regulating the expression of key genes/associated loci by MECP2, which is a DNA methylation protein implicated in Rett syndrome (Amir et al., 1999), autism (Loat et al., 2008), mental retardation (Shahbazian and Zoghbi, 2001), and mild learning disabilities (Shibayama et al., 2004). MECP2 has been classically defined as a suppressor of transcription due to its binding to methylated CpG dinucleotide, resulting in tighter winding of the chromatin coil and reduced transcription (Joyner et al., 2009). A MECP2 haplotype was shown to be associated with reduced cortical surface area in humans in the Alzheimer’s Disease Neuroimaging Initiative (ADNI) study (Joyner et al., 2009). Defective MECP2 function has also been implicated in neural tube defect (Petazzi et al., 2014), abnormal spine dynamics (Jiang et al., 2013), impaired neuronal excitability (Zhang et al., 2014), and oxidative brain damage (De Felice et al., 2014). Although the overall number of significantly altered genes were minimal in hTau^*MaptKO*(*Duke*)^ compared to WT mice, some of them have been implicated in human PSP/CBD. Finally, a previous study attempted to perform cell specific micro-array analysis in the HTau mice via micro-aspiration of CA1 pyramidal neurons by laser capture microdissection and custom micro-array analysis of 576 genes (see Supplementary Figure S7). Approximately, 8% (42 genes) of the genes were differentially regulated in the CA1 neurons from Htau mice compared to controls and these genes were associated with decreased synaptic function and neuron survival (see Supplementary Figure S7). We selected these 42 differentially regulated genes (identified in Alldred et al., 2012) from our normalized whole genome gene expression data sets and plotted for WT and hTau^*MaptKO*(*Duke*)^ mice. As expected, mouse *Mapt* was significantly higher in the WT mice compared to hTau^*MaptKO*(*Duke*)^ mice (Supplementary Figure S7). Interestingly, some of the genes (for example *Arc*) that were down-regulated in Alldred et al. (2012) study also appeared to be slightly down-regulated in the hTau^*MaptKO*(*Duke*)^ mice (Supplementary Figure S7).

Metacore^TM^-based network analysis identified SP1 transcription factor as one of the principle transcription factors regulating nine (out of 64) differentially altered genes in the hTau^*MaptKO*(*Duke*)^ mice. Notably, SP1 transcription factor responds to inflammatory signals and has been shown to be up-regulated in human AD brain, as well as in the brains of mouse models of AD (Citron et al., 2008). In a recent study, pharmacological inhibition of SP1 in a mouse model of AD resulted in accelerated amyloid pathology and impaired cognitive function (Citron et al., 2015). Finally, various neuronal pro-survival factors (Bcl-2, Bcl-x, and surviving) have been shown to be targets of SP1 (Ryu et al., 2003), suggesting the need for in-depth analysis of the role of SP1 in tauopathies.

## Conclusion

We report here on hTau^*MaptKO*(*Duke*)^ mice as a novel humanized mouse model of tauopathy with complete deficiency of endogenous mouse *Mapt*. The hTau^*MaptKO*(*Duke*)^ mice display tau hyperphosphorylation on multiple serine and threonine residues relevant to AD/tauopathies. Whole genome gene expression analysis revealed a limited number of genes differentially altered in hTau^*MaptKO*(*Duke*)^ mice when the FDR cut-off was stringent. Upon relaxation of the criteria and consideration of raw significance of *p* < 0.001, 64 genes were found to be differentially expressed in the hippocampus of hTau^*MaptKO*(*Duke*)^ mice compared to non-transgenic control mice. Importantly, SP1 transcription factor and MECP2 were identified as two key regulators implicated in hTau^*MaptKO*(*Duke*)^ mice. Notably, siRNA-mediated deficiency of MECP2 resulted in reduced total and phosphorylated tau levels in human tau expressing N2a cell culture model of tauopathy. Together, our results suggest that MECP2 may be a key regulator of tauopathy and future studies exploring the relationship between MECP2 and tau is important in understanding the molecular mechanisms of tauopathy.

## Availability of Data and Material

Microarray data are available in the ArrayExpress database (www.ebi.ac.uk/arrayexpress) under accession number E-MTAB-5078. All other data needed to evaluate the conclusions in the paper are present in the paper. Any additional data can be made available from authors upon request. Materials that are allowed for sharing can be obtained through an MTA.

## Author Contributions

KB and BL designed the study and wrote the manuscript. KB performed immunohistochemical analysis, analyzed, and interpreted the data. BG and CW performed genomic and network analysis. NM performed western blot analysis, quantifications and drafted the manuscript. SJ and JB performed *in vitro* cell culture studies and assisted with manuscript preparation.

## Conflict of Interest Statement

The authors declare that the research was conducted in the absence of any commercial or financial relationships that could be construed as a potential conflict of interest.

## Abbreviations

ABC: avidin:biotinylated enzyme complex
AD: Alzheimer’s disease
ANOVA: analysis of variance
CBD: corticobasal degeneration
CpG: 5′-C—phosphate—G-3′ (cytosine and guanine separated by only one phosphate)
DAB: 3,3’-diaminobenzidine tetrahydrochloride
EGFP: enhanced green fluorescent protein
ETS: ETS proto-oncogene 1, transcription factor
FDR: false discovery rate
FN3K (*Fn3k*): fructosamine 3 kinase
FOXK2 (*Foxk2*): forkhead box K2
FTDP-17: fronto-temporal dementia and parkinsonism linked to chromosome 17
GAPDH: glyceraldehyde 3-phosphate dehydrogenase
GH (*Gh or Gh1*): growth hormone or growth hormone 1
GO: gene ontology
IF: immunofluorescence
IHC: immunohistochemistry
IPA^TM^: Ingenuity Pathway Analysis
KRT12 (*Krt12*): type I intermediate filament chain keratin 12
LASS1 (*Cers1*): ceramide synthase 1
MAPT (*Mapt*): microtubule-associated protein tau
MECP2 (*Mecp2*): methyl-CpG-binding protein 2
MED7 (*Med7*): mediator complex subunit 7
NFT: neurofibrillary tangles
NFκB: nuclear factor kappa-light-chain-enhancer of activated B cells
NRXN1 (*Nrxn1*): neurexin 1
p-PKCα: phosphorylated PKCα
PBST: phosphate buffer saline with 0.1% tween
PCSK2 (*Pcsk2*): proprotein convertase subtilisin/kexin type 2
PiD: Pick’s disease
PKC (*Prkca*): protein kinase C alpha
pMECP2: phosphorylated MECP2
POLD2 (*Pold2*): polymerase (DNA) delta 2, accessory subunit
PSP: progressive supranuclear palsy
PXR: pregnane X receptor
QC: quality control
qRT-PCR: quantitative reverse transcription polymerase chain reaction
R-LIMMA: linear models for microarray and RNA-sequence
RAPGEFL1 (*Rapgefl1*): rap guanine nucleotide exchange factor like 1
RAR/RXR: retinoic acid receptors/retinoid receptor
SDS-PAGE: sodium dodecyl sulfate polyacrylamide gel electrophoresis
SFK: Src family kinase
SLC40a1 (*Slc40a1*): solute carrier family 40 member 1
STRN4 (*Strn4*): striatin 4
T-MECP2: total MECP2
T-PER: tissue protein extraction reagent
T-PKCα: total PKCα
TSC22D3 (*Tsc22d3*): TSC22 domain family member 3
VAT1L (*Vat1l*): vesicle amine transport 1-like
WDR18 (*Wdr18*): WD repeat domain 18
WG6: whole genome 6
WT: wild type
YBX3 (*Ybx3*): Y-box binding protein 3
