# Supplementary material for: Whole Genome Expression Analysis in a Mouse Model of Tauopathy Identifies MECP2 as a Possible Regulator of Tau Pathology
Source: Front Mol Neurosci. 2017 Mar 17;10:69. doi: 10.3389/fnmol.2017.00069 (PMC5355442; doi:10.3389/fnmol.2017.00069)
Supplement: Supplementary file 1 [file Data_Sheet_1.pdf]

Supplementary Material

Whole genome expression analysis in a mouse model of tauopathy identifies MECP2 as a possible regulator of tau pathology

Nicole Maphis[1], Shanya Jiang[1], Jessica Binder[1], Carrie Wright[2], Banu Gopalan[3], Bruce T. Lamb[5] and Kiran Bhaskar[1§]

*** Correspondence:** Kiran Bhaskar, Ph.D.: kbhaskar@salud.unm.edu

Supplementary Data

1. Supplementary Table 1
2. Supplementary Figure Legends
3. Supplementary References
4. Supplementary Figures S1 – S7

1. Supplementary Table 1: List of differentially altered genes, their names, primary function and relevance to CNS diseases.

Significantly up-regulated genes			
**Gene**	**Name**	**Primary function**	**Relevance to brain disease (source: GeneCards®)**
Prkca	Protein Kinase C-alpha	Serine/Threonine protein kinase activated by calcium and the second messenger diacylglycerol.	PKC family members phosphorylate a wide variety of protein targets and are known to be involved in diverse cellular signaling pathways.
Strn4	Striatin, calmodulin binding protein 4	Binds to calmodulin and protein phosphatase 2A	STRN4 is associated with cerebral cavernous malformations 3, and cavernous malformation
Mecp2	Methyl CpG Binding protein 2	Binds to methylated DNA and suppress gene expression, especially during neural maturation.	MECP2 gene mutations or duplication causes Rett syndrome (common cause of mental retardation in females). Loss of MECP2 causes neurological disorder (Petazzi et al., 2014). SNP in *MECP2* gene show association with reduced cortical surface area in AD (a study using ADNI samples) (Joyner et al., 2009)
Slc40a1	Solute carrier family 40 (iron-regulated transporter), member 1	Cellular iron transporter	Reduced in the peripheral bone marrow macrophages in human AD subjects. Single nucleotide polymorphism allele shows association to AD. (Crespo et al., 2014)
PolD2	Polymerase (DNA directed), Delta 2,	This gene encodes the 50-kDa catalytic subunit of DNA polymerase delta. DNA polymerase delta	Gliomas (Bredel et al., 2009)

	accessory subunit	possesses both polymerase and 3' to 5' exonuclease activity and plays a critical role in DNA replication and repair. The encoded protein is required for the stimulation of DNA polymerase delta activity by the cofactor proliferating cell nuclear antigen (PCNA). Expression of this gene may be a marker for ovarian carcinomas.	
Fn3k	Fructoseamine 3 kinase	Catalyzes the phosphorylation of fructosamines which may result in deglycation	Hyperglycemia, and diabetes mellitus.
Pcsk2	Proprotein convertase subtilisin/Kexin type 2	This gene encodes a member of the subtilisin-like proprotein convertase family, which includes proteases that process protein and peptide precursors trafficking through regulated or constitutive branches of the secretory pathway.	Diseases associated with PCSK2 include neuroepithelioma, and glucagonoma.
Rps7	Ribosomal protein S7	Encodes a ribosomal protein that is a component of the 40S subunit.	Diamond-blackfan anemia 8, and rps7-related diamond-blackfan anemia
Ino80c	INO80 Complex Subunit C	INO80 complex is involved in transcriptional regulation, DNA replication and repair	Malaria

**Significantly down-regulated genes**			
Krt12	Keratin 12	Type 1 intermediate filament chain keratin 12 expressed in corneal epithelia	Meesmann corneal dystrophy and recurrent corneal erosion
Lass1/Cers1	Ceramide Synthase 1	Encodes a member of the bone morphogenetic protein (BMP) family and the TGF-beta superfamily.	Mental retardation and cerebritis
Plat	Plasminogen activator, tissue	Encodes for a secreted serine protease which converts the proenzyme plasminogen to plasmin – a fibrinolytic enzyme	Pulmonary embolism, venous thoracic outlet syndrome. Single nucleotide polymorphism show association to late-onset sporadic AD (Shibata et al., 2007)
Nrxn1	Neurexin 1	Cell adhesion	Pitt-Hopkins-like syndrome 2 and schizophrenia. Lower copy number variation in NRXN1 show association to human AD (Swaminathan et al., 2012)

2. Supplementary Figure Legends:

Supplementary Figure S1. Box-plot (microarray intensity) and line graphs (density plot of intensity) showing quality control analysis of the normalized values from six samples (hippocampus from six-month-old non-transgenic and hTau$^{MaptKO(Duke)}$ mice, n=3 per genotype).

Supplementary Figure S2. Line graphs showing the intensity (log_2) for various normalized controls as a part of quality control analysis for all six samples used in the current study.

Supplementary Figure S3. M (log ratios) and A (mean average) scale plot or Bland-Altman plot of normalized values as a part of quality control analysis for all six samples used in the current study.

Supplementary Figure S4. Pairwise plot showing sample correlations for all six samples used in the present study.

Supplementary Figure S5. Clustering plot and principal component analysis showing sample relations for all six samples used in the present study.

Supplementary Figure S6. Heat-map showing clustering of non-transgenic wild-type (WT_1-3) and hTau_1-3 (hTauMaptKO(Duke)) samples and relative expression of important human genes (*PAXIP1, SOS1, DYSF, EIF2AK3, STX6, ABCA7, MOBP* and *MAPT*) that were recently discovered as candidate genes with strong association to PSP, CBD and PiD in recent GWAS studies.

Supplementary Figure S7. Heat-map showing clustering of non-transgenic (WT_1-3) and hTau$^{MaptKO(Duke)}$ (hTau_1-3) samples and relative expression of 42 differentially expressed genes. These genes were reported to be differentially expressed in the laser-capture micro-aspirated CA1 hippocampal neurons from 11-14 month old HTau mice compared to age-matched non-transgenic controls (from a previously published study (Alldred et al., 2012)). We separated these genes from our array and plotted to compare their expression in WT and hTau$^{MaptKO(Duke)}$ samples. Note that the highly expressed genes are shown in red (for example: *Syp* or synaptophysin) and lowest expressing genes are shown in green (example: *Rgs13* or regulator of G-protein signaling 13). Also note that mouse *Mapt* (mouse tau) is markedly reduced in hTau$^{MaptKO(Duke)}$ mice (greener signal in hTau_1-3), but present in WT_1-3 (closer to redness) and the *Arc* (Activity Regulated Cytoskeleton Associated Protein) appears to be slightly down-regulated in hTau_1-3 compared to WT_1-3 – an observation also published in (Alldred et al., 2012)).

3. Supplementary References:

Alldred, M.J., Duff, K.E., and Ginsberg, S.D. (2012). Microarray analysis of CA1 pyramidal neurons in a mouse model of tauopathy reveals progressive synaptic dysfunction. *Neurobiol Dis* 45(2), 751-762. doi: 10.1016/j.nbd.2011.10.022.

Bredel, M., Scholtens, D.M., Harsh, G.R., Bredel, C., Chandler, J.P., Renfrow, J.J., et al. (2009). A network model of a cooperative genetic landscape in brain tumors. *JAMA* 302(3), 261-275. doi: 10.1001/jama.2009.997.

Crespo, A.C., Silva, B., Marques, L., Marcelino, E., Maruta, C., Costa, S., et al. (2014). Genetic and biochemical markers in patients with Alzheimer's disease support a concerted systemic iron homeostasis dysregulation. *Neurobiol Aging* 35(4), 777-785. doi: 10.1016/j.neurobiolaging.2013.10.078.

Joyner, A.H., J, C.R., Bloss, C.S., Bakken, T.E., Rimol, L.M., Melle, I., et al. (2009). A common MECP2 haplotype associates with reduced cortical surface area in humans in two independent populations. *Proc Natl Acad Sci U S A* 106(36), 15483-15488. doi: 10.1073/pnas.0901866106.

Petazzi, P., Akizu, N., Garcia, A., Estaras, C., Martinez de Paz, A., Rodriguez-Paredes, M., et al. (2014). An increase in MECP2 dosage impairs neural tube formation. *Neurobiol Dis* 67, 49-56. doi: 10.1016/j.nbd.2014.03.009.

Shibata, N., Kawarai, T., Meng, Y., Lee, J.H., Lee, H.S., Wakutani, Y., et al. (2007). Association studies between the plasmin genes and late-onset Alzheimer's disease. *Neurobiol Aging* 28(7), 1041-1043. doi: 10.1016/j.neurobiolaging.2006.05.028.

Swaminathan, S., Shen, L., Kim, S., Inlow, M., West, J.D., Faber, K.M., et al. (2012). Analysis of copy number variation in Alzheimer's disease: the NIALOAD/ NCRAD Family Study. *Curr Alzheimer Res* 9(7), 801-814.

Supplementary Figure S1

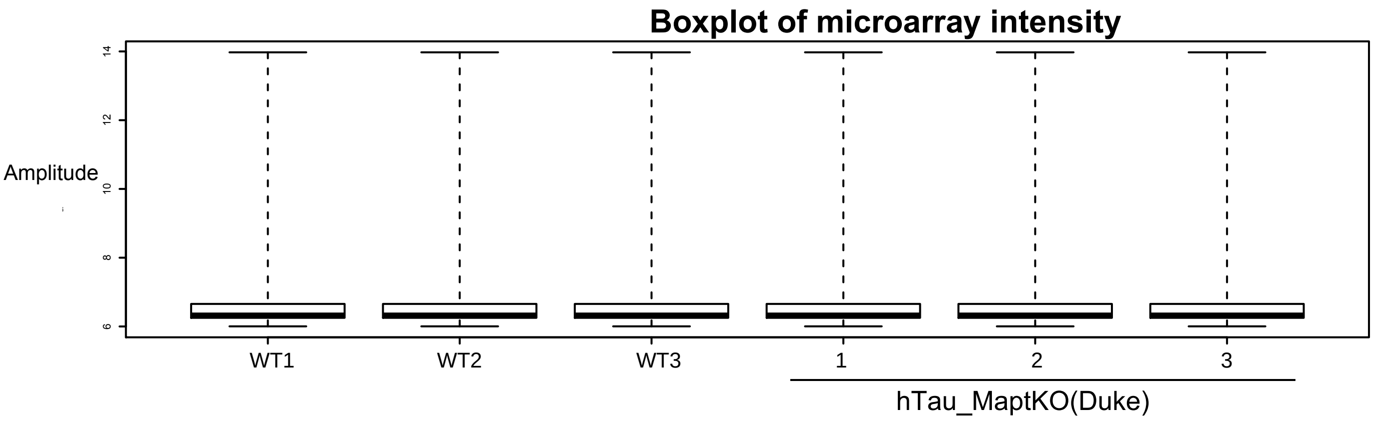

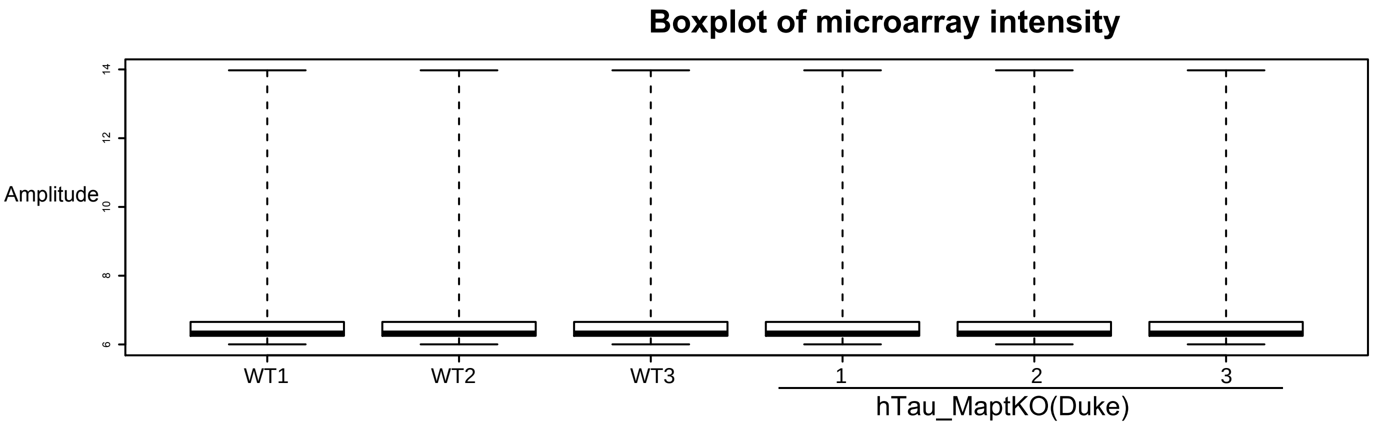

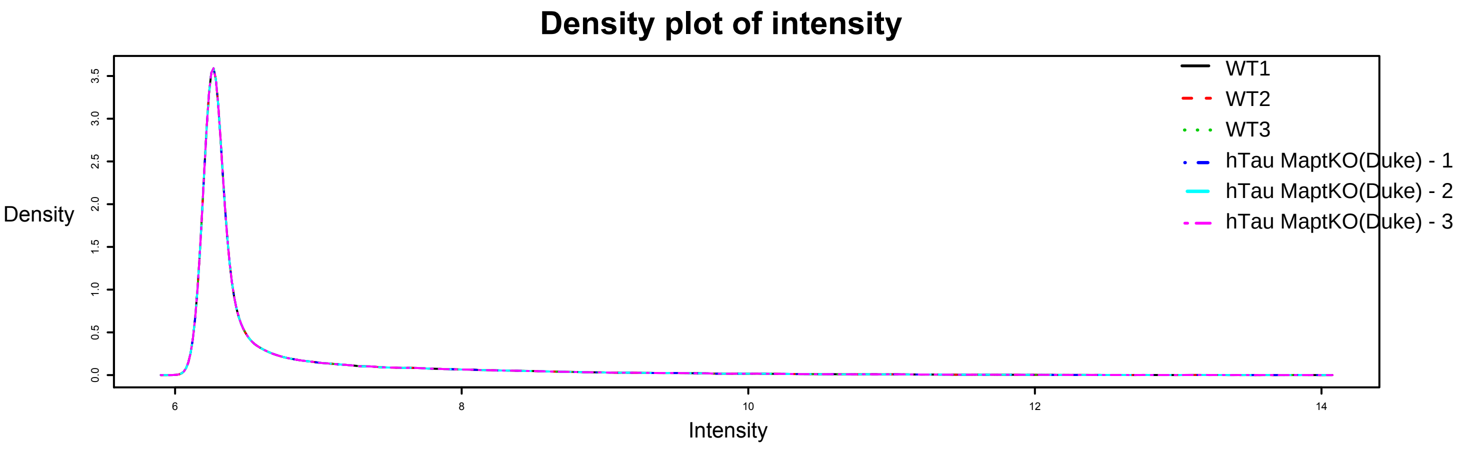

Supplementary Figure S2

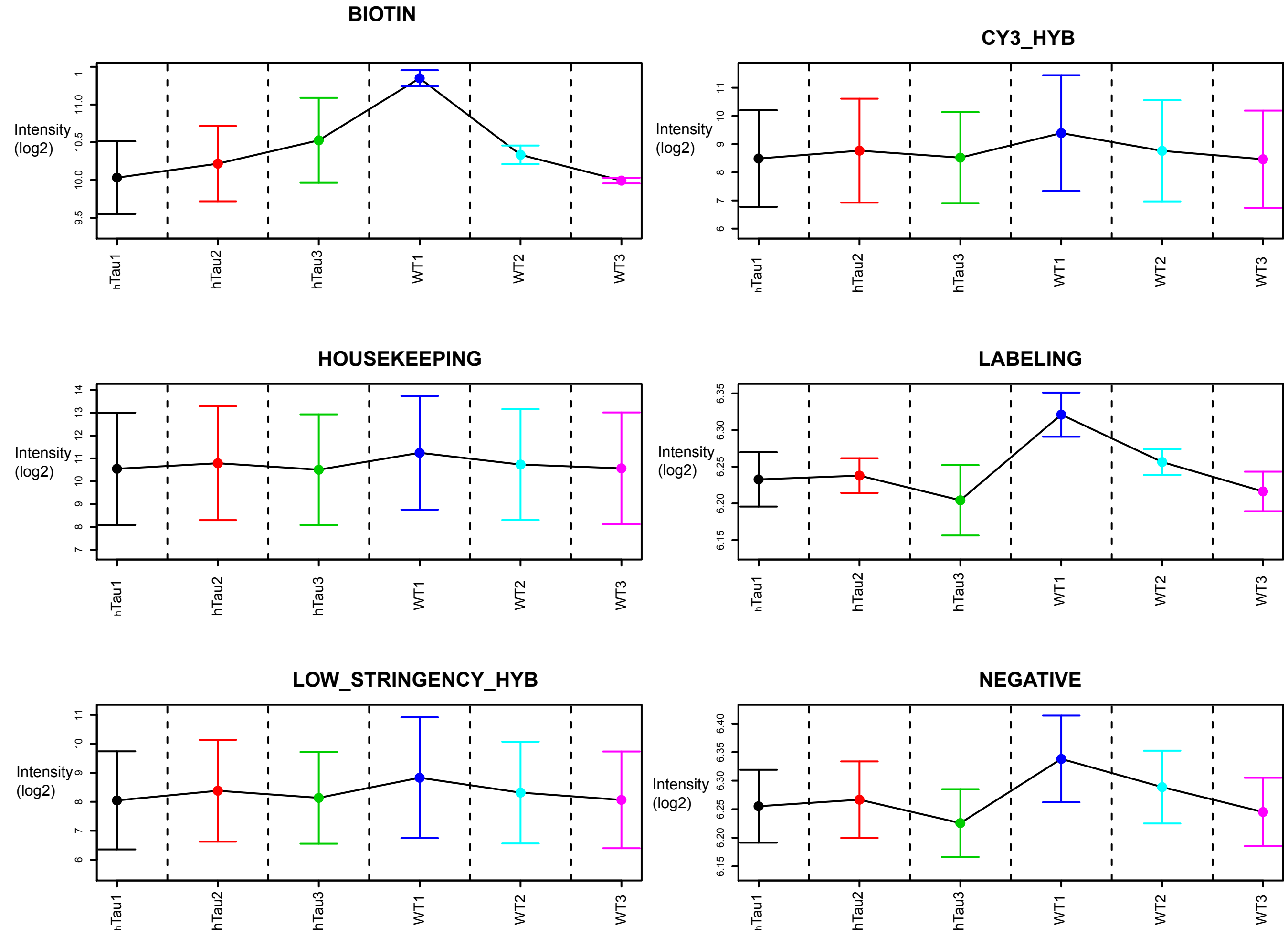

Supplementary Figure S3

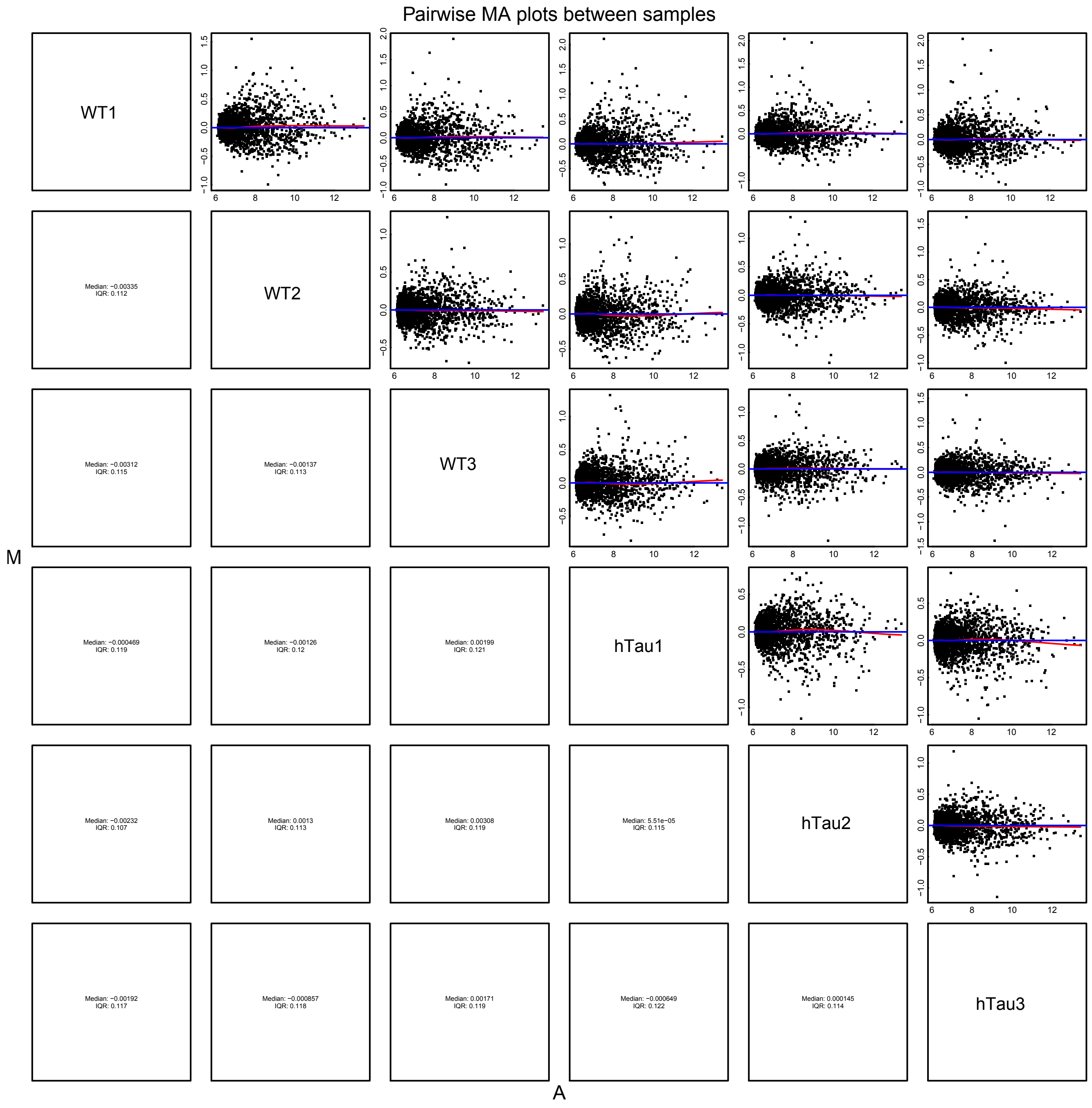

Supplementary Figure S4

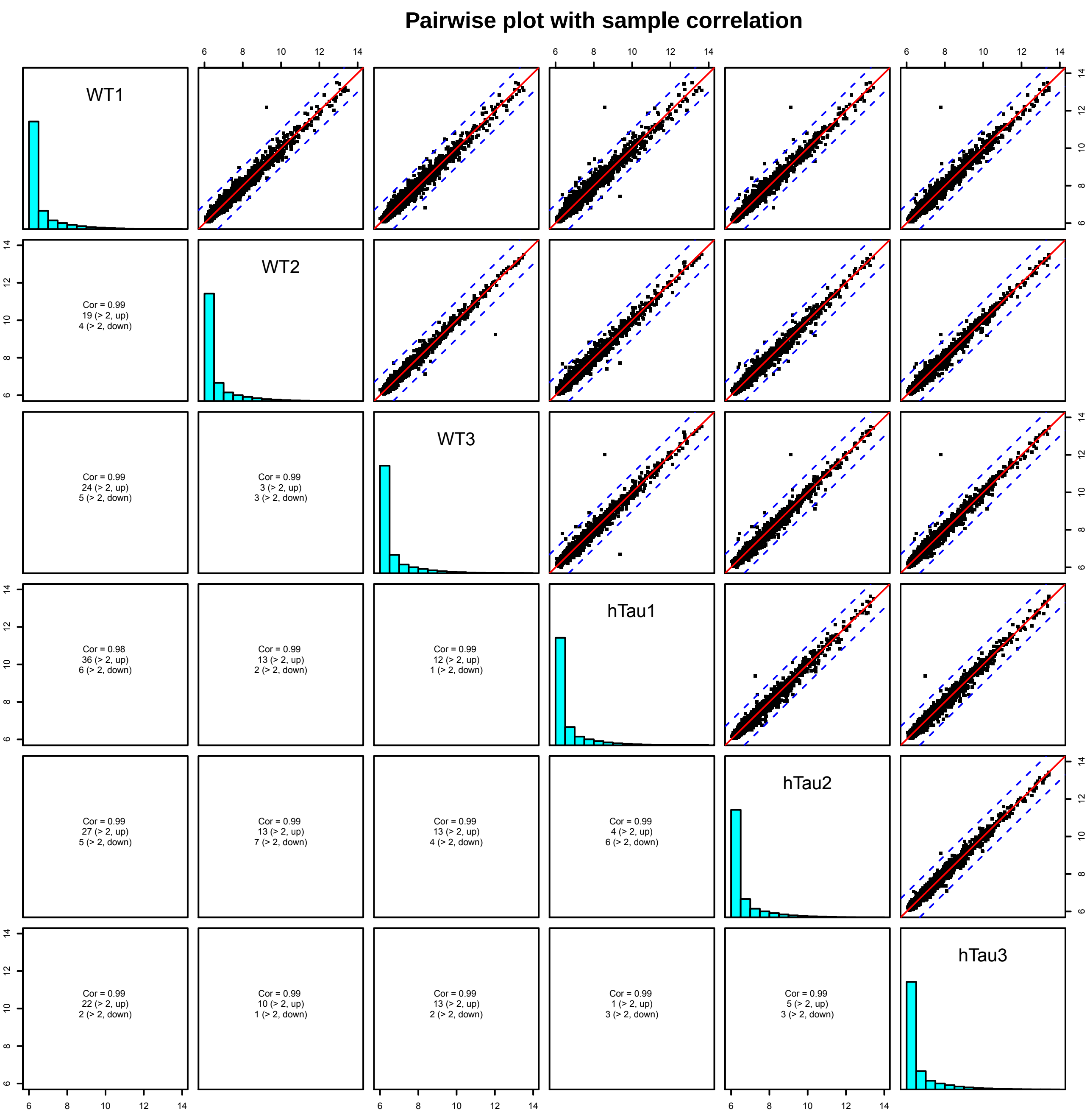

Supplementary Figure S5

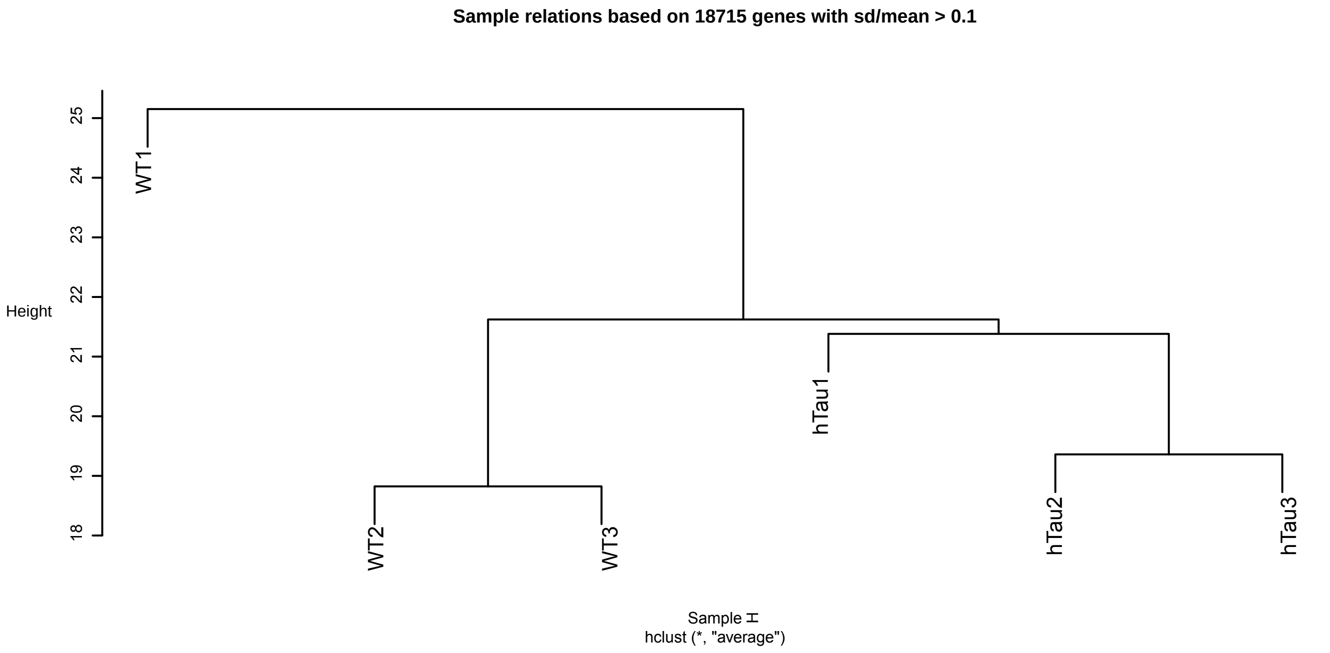

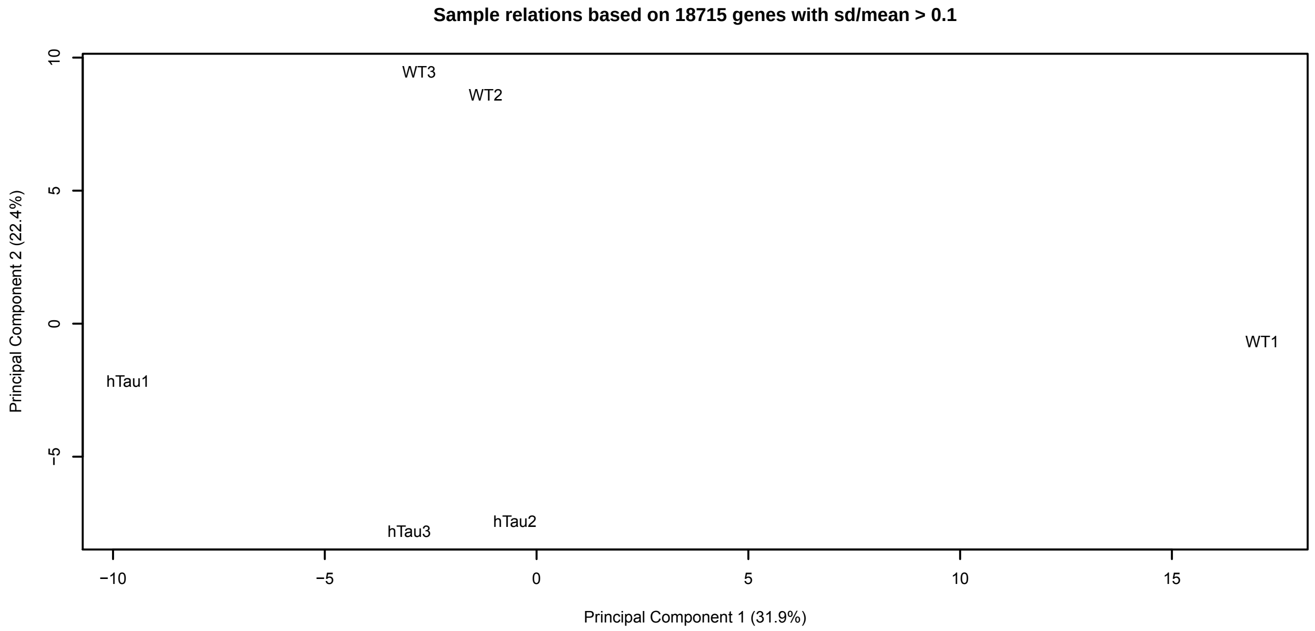

Supplementary Figure S6

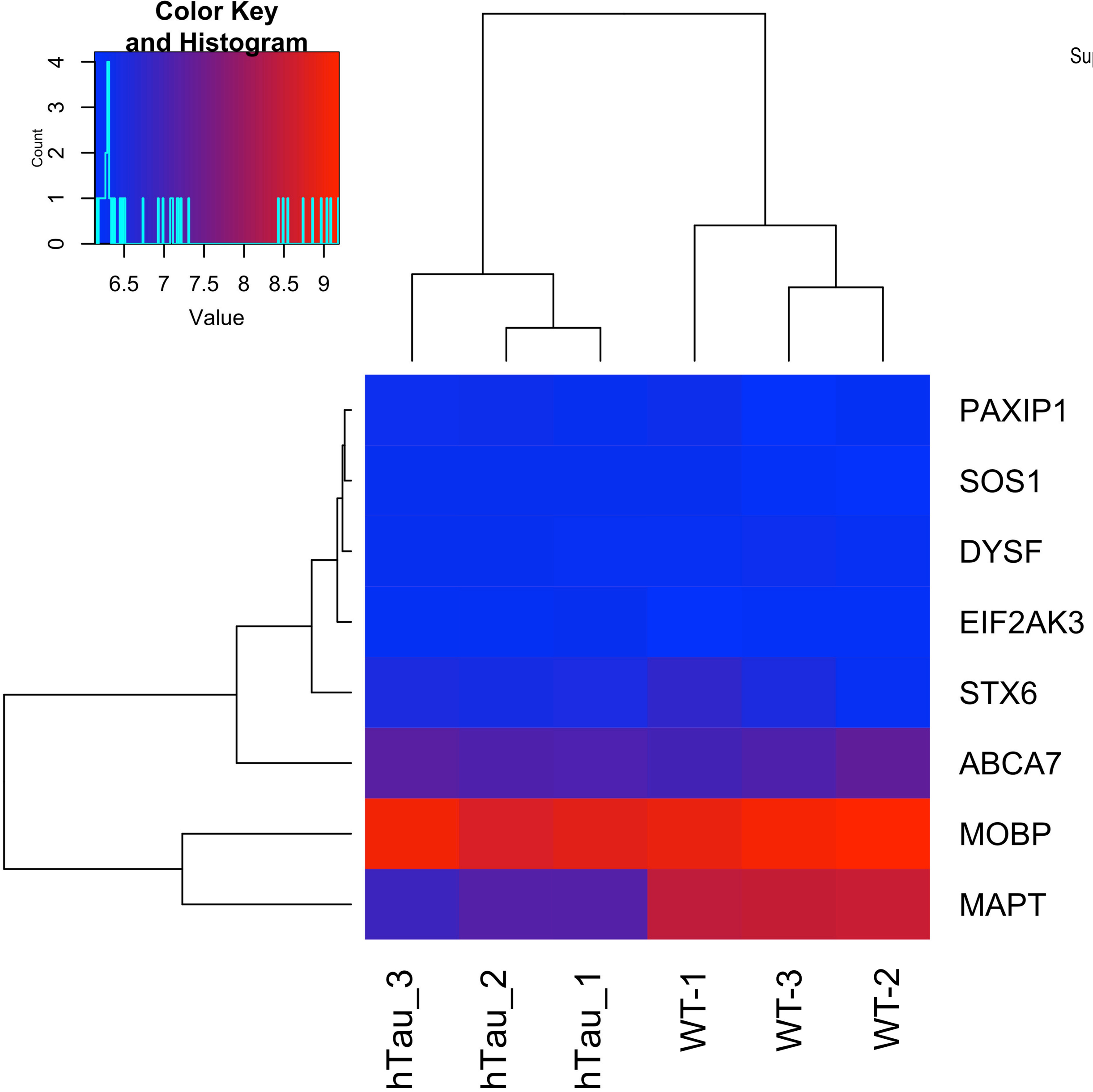

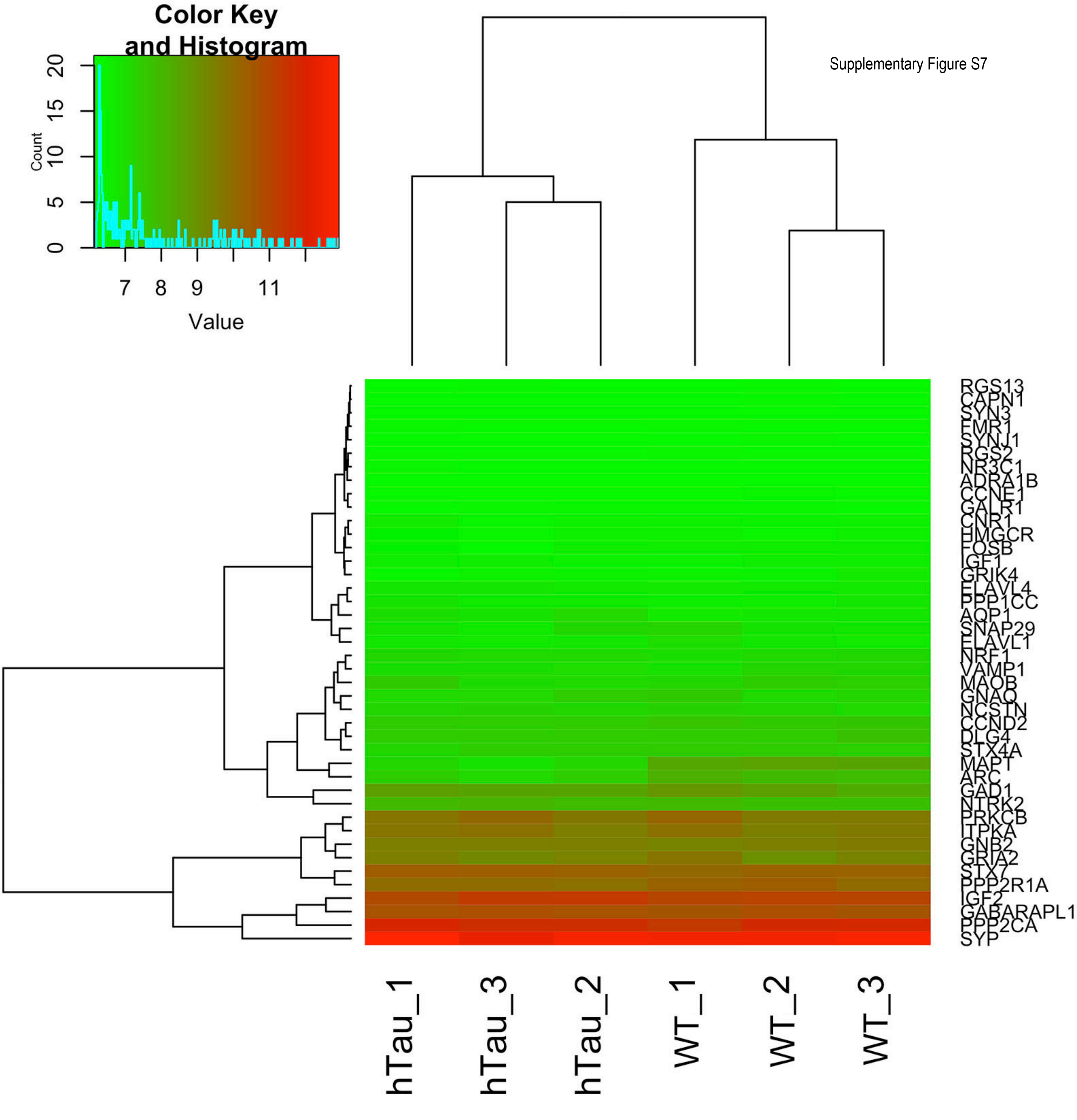

Supplementary Figure S7
